# Influence of Digital Intervention Messaging on Influenza Vaccination Rates Among Adults With Cardiovascular Disease in the United States: Decentralized Randomized Controlled Trial

**DOI:** 10.2196/38710

**Published:** 2022-10-07

**Authors:** Nell J Marshall, Jennifer L Lee, Jessica Schroeder, Wei-Nchih Lee, Jermyn See, Mohammad Madjid, Mrudula R Munagala, John D Piette, Litjen Tan, Orly Vardeny, Michael Greenberg, Jan Liska, Monica Mercer, Sandrine Samson

**Affiliations:** 1 Evidation Health, Inc San Mateo, CA United States; 2 David Geffen School of Medicine University of California Los Angeles, CA United States; 3 Department of Cardiology University of Miami Miami, FL United States; 4 Department of Health Behavior and Health Education School of Public Health University of Michigan Ann Arbor, MI United States; 5 Immunize.org St. Paul, MN United States; 6 Center for Care Delivery and Outcomes Research Veterans Health Administration Minneapolis, MN United States; 7 Sanofi Swiftwater, PA United States; 8 Sanofi Gentilly France; 9 Sanofi Lyon France

**Keywords:** influenza, randomized trial, public health, cardiovascular disease, immunization, vaccination, digital messaging, digital intervention, mobile health, mHealth

## Abstract

**Background:**

Seasonal influenza affects 5% to 15% of Americans annually, resulting in preventable deaths and substantial economic impact. Influenza infection is particularly dangerous for people with cardiovascular disease, who therefore represent a priority group for vaccination campaigns.

**Objective:**

We aimed to assess the effects of digital intervention messaging on self-reported rates of seasonal influenza vaccination.

**Methods:**

This was a randomized, controlled, single-blind, and decentralized trial conducted at individual locations throughout the United States over the 2020-2021 influenza season. Adults with self-reported cardiovascular disease who were members of the Achievement mobile platform were randomized to receive or not receive a series of 6 patient-centered digital intervention messages promoting influenza vaccination. The primary end point was the between-group difference in self-reported vaccination rates at 6 months after randomization. Secondary outcomes included the levels of engagement with the messages and the relationship between vaccination rates and engagement with the messages. Subgroup analyses examined variation in intervention effects by race. Controlling for randomization group, we examined the impact of other predictors of vaccination status, including cardiovascular condition type, vaccine drivers or barriers, and vaccine knowledge.

**Results:**

Of the 49,138 randomized participants, responses on the primary end point were available for 11,237 (22.87%; 5575 in the intervention group and 5662 in the control group) participants. The vaccination rate was significantly higher in the intervention group (3418/5575, 61.31%) than the control group (3355/5662, 59.25%; relative risk 1.03, 95% CI 1.004-1.066; *P*=.03). Participants who were older, more educated, and White or Asian were more likely to report being vaccinated. The intervention was effective among White participants (*P*=.004) but not among people of color (*P*=.42). The vaccination rate was 13 percentage points higher among participants who completed all 6 intervention messages versus none, and at least 2 completed messages appeared to be needed for effectiveness. Participants who reported a diagnosis of COVID-19 were more likely to be vaccinated for influenza regardless of treatment assignment.

**Conclusions:**

This personalized, evidence-based digital intervention was effective in increasing vaccination rates in this population of high-risk people with cardiovascular disease.

**Trial Registration:**

ClinicalTrials.gov NCT04584645; https://clinicaltrials.gov/ct2/show/NCT04584645

## Introduction

About 5% to 15% of the US population contracts influenza annually [1], resulting in more than 20,000 deaths [1] and substantial economic impact [2]. For people with cardiovascular disease (CVD), influenza can be particularly dangerous. In one study, the risk of myocardial infarction was 6 times higher within a week of influenza infection [3]. A study of more than 80,000 US adults hospitalized with influenza over 8 seasons found that 1 in every 8 patients developed sudden, serious cardiac complications and that having underlying cardiac disease was significantly associated with experiencing an acute cardiac event with influenza [4]. For these reasons, the Centers for Disease Control and Prevention (CDC) consider persons with CVD to be at high risk for influenza complications and therefore a priority group for vaccination [5].

Vaccination remains the most effective primary prevention method against influenza, with age-adjusted effectiveness rates of up to 68% over the past 5 years [6]. The CDC reported a 51.4% vaccination rate for 2019-2020 for persons aged 18-64 years who have high-risk conditions such as CVD [7], far below the 70% national vaccination rate goal [8]. Given the increased burden of influenza for people with CVD, even small improvements in vaccination rates could substantially reduce the number of patients having major adverse cardiac events [2,9-11].

Novel, scalable, cost-optimal, and effective solutions are needed to address barriers to influenza vaccination among people with CVD, such as complacency, time and cost constraints, and a lack of confidence [12]. Observational [13] and randomized controlled trials [14,15] have shown the effectiveness of digital messaging to increase vaccine uptake in general adult populations. In a randomized trial of digital messaging in persons with diabetes, a population also at increased risk of influenza-related complications [16], the vaccination rate was 3.1% higher in the intervention group than the control group. Alternatively stated, 33 people would need to receive the intervention for 1 additional person to become vaccinated.

The primary objective of this study was to examine the efficacy of a digital intervention designed to increase self-reported influenza vaccination rates in individuals with CVD.

## Methods

### Study Design

This 8-month, pragmatic randomized controlled trial was conducted remotely in the United States. Participants were blinded to study participation status to minimize observation bias, although all participants agreed that their survey responses and behavioral data would be used for research purposes before completing each survey (see below).

All participants were members of the free Achievement mobile health and research platform (Evidation Health, Inc), which includes more than 4 million individuals spanning all 50 states and 90% of zip codes [17]. The platform provides personalized insights and tools to motivate and empower people to take evidence-supported actions to manage their health. Members can connect activity trackers and fitness and health apps to the platform and share self-reported health information. Achievement does not have the ability to access clinical or claims data; it relies solely on member-generated data.

### Ethics Approval

The trial protocol was approved by Solutions IRB, Yarnell, Arizona (Registration: IORG0007116; Federalwide Assurance: IRB00008523), and registered at ClinicalTrials.gov (NCT04584645). Since the digital intervention messages were consistent with publicly available information on influenza vaccination, we obtained a waiver of informed consent from this Institutional Review Board on the basis that participants would face only minimal risk from the study. Participants were informed about how their survey responses and behavioral data would be used through a Data Usage and Permissions Agreement.

### Digital Intervention Design

The 6 digital intervention messages were developed using a 3-part approach [18], building on a previous study [16] and the Theory of Planned Behavior [19]. Message designs were refined throughout the development process using Rapid Iterative Testing and Evaluation–inspired methods [20]. See Multimedia Appendix 1 [16,18-21] for details of the development process and the content of the intervention messages (Table S1 and Figures S1-7 in Multimedia Appendix 1), which were delivered via the Achievement platform.

### Participants

Eligible participants were those aged ≥18 years, living in the United States, with any of the following self-reported conditions on the Achievement platform (eg, through past surveys): atrial fibrillation; abnormal or irregular heart rhythm or other arrhythmic heart disease; cardiac arrest or myocardial infarction; coronary artery disease treated with medication, stenting, percutaneous intervention, or bypass surgery; congestive heart failure; or stroke or cerebrovascular accident.

### Recruitment, Screening, and Enrolling

Members who met the inclusion criteria were identified for study inclusion (“participants”). Participants took no action to enroll and were not informed about their participation status. We used block randomization by cardiovascular condition to randomize participants into either the intervention group, which received the digital intervention messages, or the control group, which received none of the messages.

### Randomization and Blinding

Evidation Health, Inc generated the random allocations, enrolled participants, and randomized them using block randomization (arrhythmia vs nonarrhythmia) into the intervention or control group before offering the opportunity to complete any study activities.

### Study Procedures

Participants were asked to complete the web-based surveys at baseline, 3 months (after 4 digital messages had been sent in the intervention group), and 6 months (after 2 more messages had been sent in the intervention group). Reminder messages were used to motivate survey completion.

### Primary and Other Outcomes

Participants self-reported their vaccination status (yes or no) via the app at baseline, 3 months, and 6 months. Participants also reported the estimated date of vaccination, if any, on the 3- and 6-month surveys.

To assess engagement with the intervention messages, we examined platform-generated data indicating that the person had completed a given message and created a summary measure indicating the number of messages completed.

Each survey measured the drivers and barriers to vaccination as well as vaccine knowledge. The vaccine drivers and barriers of interest included the number of visits to a primary care provider in the 3 months before randomization (none, 1-2, or 3 or more), number of visits to a cardiology specialist in the prior 3 months (none, 1-2, or 3 or more), number of hospitalizations in the prior 3 months (none, 1, or 2 or more), whether a health care provider had offered influenza vaccination (yes, no, or unsure), and whether a health care provider had informed the individual that they were in a “high-risk group” (yes, no, or unsure).

Vaccine knowledge factors were based on responses to the survey question “What sources of information do you use to learn about the flu vaccine?” with possible responses of health care professionals, family member or peers, social media including blog posts, mobile apps, or conventional news media (eg, television and newspapers).

### Sample Size Calculation

The sample size was determined a priori for a 2-arm interventional statistical superiority study design with self-reported vaccination rates as the primary outcome [22]. Large studies on the impact of messaging and telephone reminders to improve influenza vaccination rates show a range of effect sizes from 2.5% to 3.5% [23,24]. A total of 8000 participants were needed to detect a 3% difference in vaccination rates with a type I error rate of 0.05 and power of 0.80. Since a participation drop-off of 67% was observed for digital interventions aimed at increasing influenza vaccination in people with diabetes [16], we conservatively estimated an engagement rate of about 16%. The targeted enrollment list therefore included approximately 49,000 individuals to yield the analysis population of 8000 participants.

### Statistical Analysis

We first compared the unadjusted proportions of participants reporting vaccination at follow-up between the intervention and control groups. In predefined subgroup analyses, we examined variations in intervention effects between White and non-White participants. Process analyses included differences in self-reported vaccination rates within the intervention group by the number of intervention messages completed and intervention participants’ levels of engagement with each message. Controlling for randomization group, we examined other predictors of vaccination status, including cardiovascular condition type, vaccine drivers or barriers, and vaccine knowledge.

An exploratory objective was to describe the impact of the COVID-19 pandemic on influenza vaccination behavior. Another exploratory objective—self-reported complications from influenza, overall and by vaccination status—was not analyzed because the surveys did not ask about influenza complications. Information on safety and adverse events was not collected, given the minimal-risk nature of the intervention and study.

Variables were compared at the 5% significance level using 2-sided tests or 2-sided 95% CI unless otherwise specified. Comparison of means used 2-sided Student *t* test for normal distributions or a Mann-Whitney *U* test for nonnormal distributions. Comparisons of frequencies used chi-square tests. For the logistic regression model, the *P* values, odds ratios (ORs), and 95% CIs associated with each of the β parameter estimates were reported. To describe the relative importance of each predictor variable, we calculated their Shapley Additive Explanation values [25]. Kaplan-Meier curves were constructed for time to influenza vaccination, using the participant-estimated dates of influenza vaccination from the 3- and 6-month surveys.

## Results

### Participants

**Figure 1:**
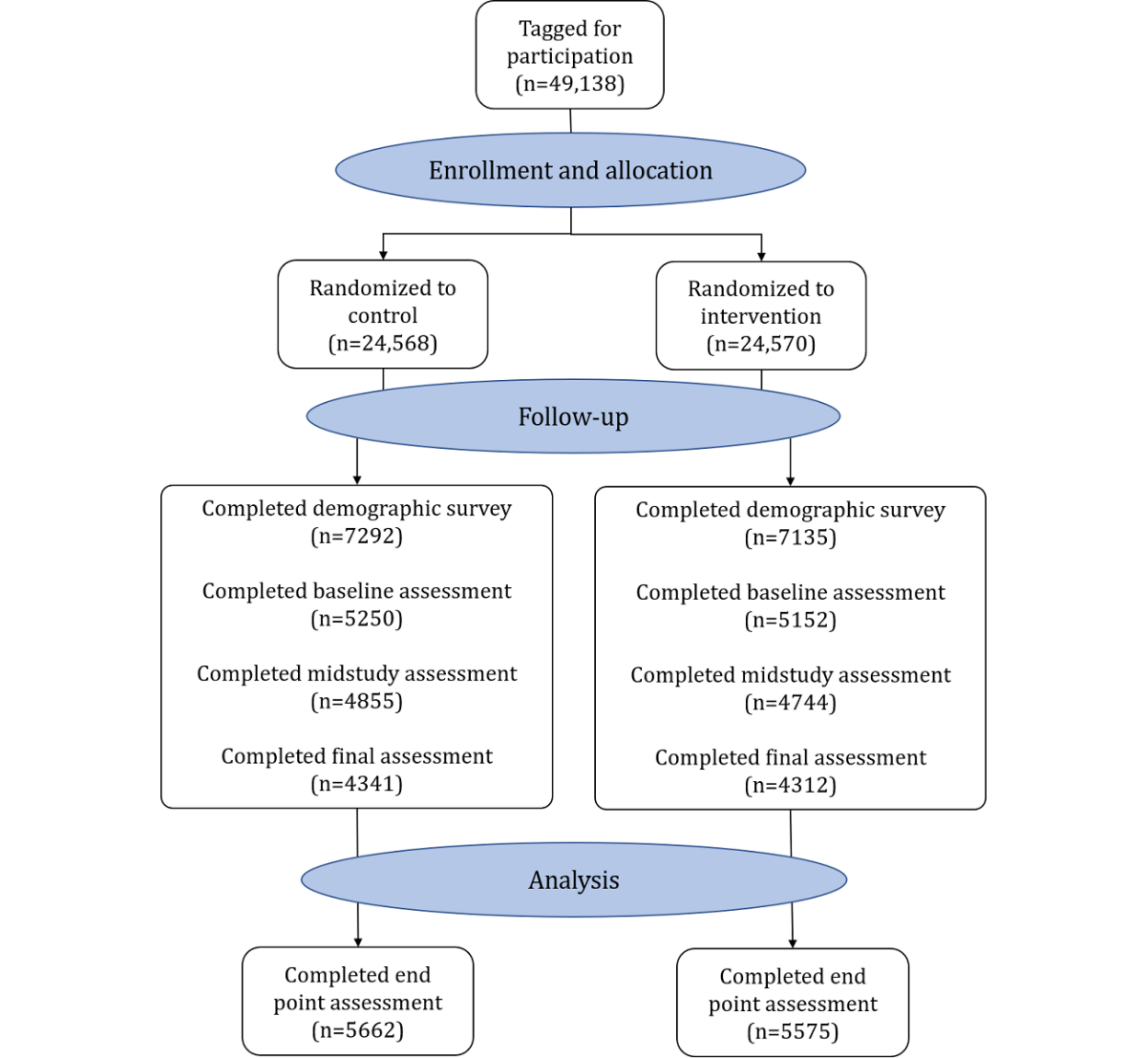
Disposition of Study Participants.

Between July and September 2020, we generated a list of 49,138 candidate participants (Figure 1). Of these, 24,570 were randomized to receive digital intervention messages and 24,568 were randomized to the control group. On September 21, 2020, the first baseline and demographic surveys were sent to these 49,138 participants, and 10,402 (21.17%) completed the baseline survey. In all, 11,237 participants (22.87%) completed the midstudy or final survey by April 11, 2021, yielding groups of 5575 intervention and 5662 control participants who reported vaccination status at either 3 or 6 months after randomization.

Of the 11,237 participants, the average age was 45 (SD 13) years, 81.18% (n=9122) were White, 78.01% (n=8766) were female, and 86.21% (n=9687) had health insurance (Table 1). More than half (n=6891, 61.32%) had a college degree, and a third (n=3770, 33.55%) had a household income of at least US $75,000. The most commonly reported cardiovascular condition was arrhythmia (intervention: 2251/5575, 40.38%; control: 2331/5662, 41.17%). Baseline characteristics did not differ substantially between groups. Despite previous self-reports of CVD from all participants, almost a third (intervention: 1798/5575, 32.35%; control: 1844/5662, 32.57%) in both groups reported not having any of the listed conditions in the baseline survey. Study participants represented all 50 states and the District of Columbia (Figure S8 in Multimedia Appendix 1).

**Table 1:** Baseline demographic and clinical characteristics of the participants.

Characteristic		Intervention (N=5575)	Control (N=5662)
Age (years; intervention: n=5530; control: n=5607), mean (SD)		45.0 (13.5)	44.9 (13.3)
**Sex, n (%)^a^**			
	Female	4339 (77.83)	4427 (78.19)
	Male	1071 (19.21)	1114 (19.68)
	Other	169 (3.03)	135 (2.38)
**Race/ethnicity, n (%)^a^**			
	American Indian or Alaska Native	155 (2.78)	143 (2.53)
	Asian	250 (4.48)	227 (4)
	Black or African American	388 (6.96)	354 (6.25)
	Hispanic, Latino, or Spanish	319 (5.72)	375 (6.62)
	Middle Eastern or North African	46 (0.83)	50 (0.88)
	Native Hawaiian or Other Pacific Islander	45 (0.81)	49 (0.87)
	White	4510 (80.9)	4612 (81.46)
	Other	60 (1.08)	64 (1.13)
	Prefer not to answer	187 (3.35)	179 (3.16)
Had health insurance, n (%)		4815 (86.37)	4872 (86.05)
Had a college degree, n (%)		3381 (60.65)	3510 (61.99)
Household income ≥US $75,000, n (%)		1861 (33.38)	1909 (33.72)
**Cardiovascular condition type, n (%)^a^**			
	Arrhythmia	2251 (40.37)	2331 (41.17)
	Atrial fibrillation	488 (8.75)	496 (8.76)
	Cardiac arrest	112 (2)	99 (1.75)
	Myocardial infarction	385 (6.91)	412 (7.28)
	Heart failure	332 (5.96)	293 (5.17)
	Coronary artery disease	366 (6.57)	356 (6.29)
	Stroke or cerebrovascular accident	433 (7.77)	436 (7.7)
	Other cardiovascular diseases	539 (9.67)	545 (9.63)
	None of the above diagnoses^b^	1798 (32.25)	1844 (32.57)

^a^Participants could choose more than 1 option, and percentages may add up to >100%.

^b^Despite previous self-reports of cardiovascular disease from all participants, some reported not having any of the included conditions at baseline. Please see the Limitations section for more details.

### Primary Outcome

By the end of the study period, 3418 (61.31%) of the 5575 participants in the intervention arm had reported obtaining influenza vaccination compared to 3355 (59.25%) of the 5662 participants in the control arm (absolute difference: 2.06%; relative risk 1.03, 95% CI 1.004-1.066; *P*=.03). Based on this difference, 48.3 persons would have to receive the digital intervention messages for 1 additional person to become vaccinated.

### Secondary Outcomes

**Figure 2:**
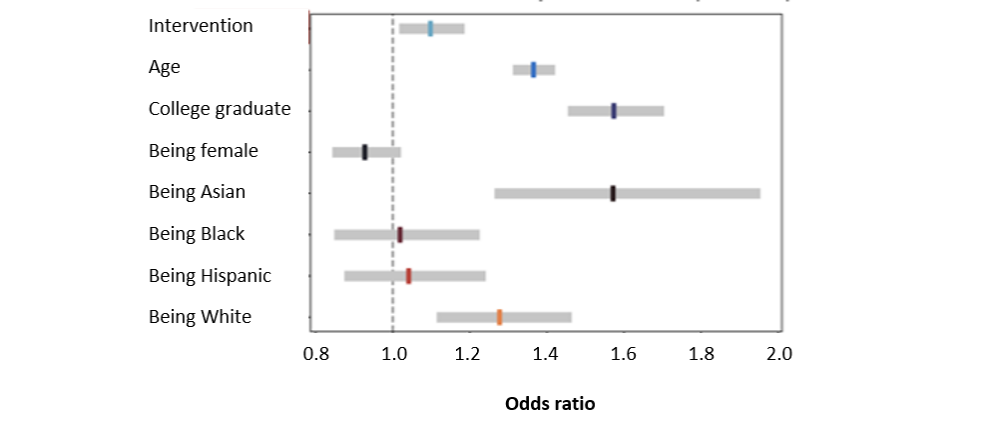
Predictors of self-reported influenza vaccination.

In logistic regression modeling, overall predictors of vaccination status included White or Asian race and being older or a college graduate (Figure 2 and Figure S9 in Multimedia Appendix 1). Being in the intervention group was associated with a significantly increased likelihood of getting the influenza vaccine (OR 1.099, 95% CI 1.012-1.192; *P*=.02). Participants who had cardiac arrest (OR 3.477, 95% CI 1.85-6.54; *P*<.001), atrial fibrillation (OR 1.332, 95% CI 1.068-1.66; *P*=.01), or coronary disease (OR 1.411, 95% CI 1.055-1.885; *P*=.02) were also more likely to report vaccination (>65%) than participants with other conditions. Digital interventions appeared to be more effective in encouraging vaccinations among White participants (intervention: 2837/4510, 62.9% vs control: 2763/4612, 59.91%; *P*=.004) than among non-White participants (intervention: 581/1065, 54.55% vs control: 593/1050, 56.48%; *P*=.42; Figure S10 in Multimedia Appendix 1).

**Figure 3:**
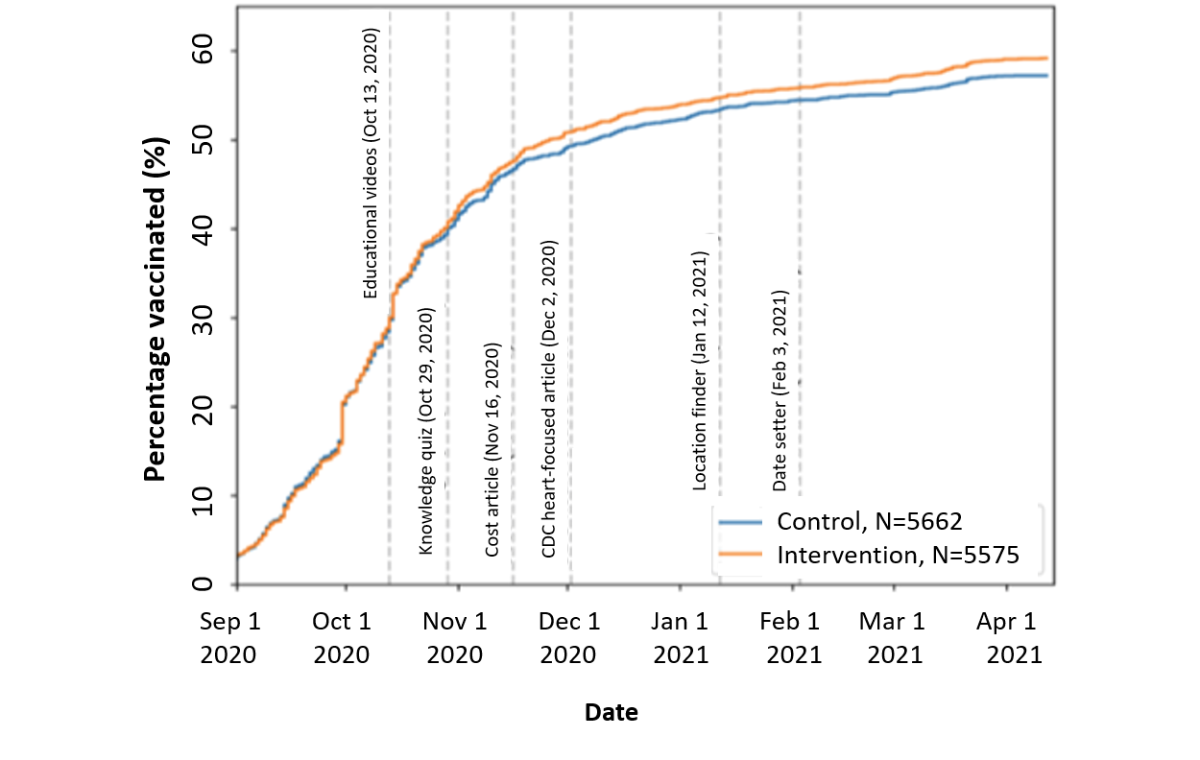
Self-reported vaccination rates over time. Dashed vertical gray lines indicate the timings of the 6 digital intervention messages. CDC: Centers for Disease Control and Prevention.

Kaplan-Meier analysis of the time to vaccination showed that at least 2 digital intervention messages, completed 2 weeks apart, were necessary for a difference to begin to emerge (Figure 3). In the intervention group (N=5575), the most completed messages were the knowledge quiz (n=4248, 76.2%), cost article (n=4276, 76.7%), and CDC article (n=4315, 77.4%; Figure S11 in Multimedia Appendix 1). In all, 44.81% (n=2498) of the intervention group completed all 6 messages, and 7.7% (n=429) completed none of them; the reported vaccination rate for the former group was about 13 percentage points higher than that for the latter group (1626/2498, 65.09% vs 223/429, 51.98%).

After controlling for age, sex, race, and education over the entire study population, participants who saw a health care provider, were offered an influenza vaccine, or got their vaccine information from a health care provider were more likely to report getting the vaccine (Figure S12 in Multimedia Appendix 1). Those who were told by a health care provider that they were part of a high-risk group were also more likely to report vaccination (OR 2.369, 95% CI 2.171-2.586; *P*<.001).

Participants who reported a diagnosis of COVID-19 were 40% more likely to report influenza vaccination than those who did not, regardless of intervention assignment (Figure S13 in Multimedia Appendix 1). Of the 7457 participants who reported getting the influenza vaccine, 4252 (57.02%) stated that the COVID-19 pandemic did not influence their decision to vaccinate, and 4026 (66.33%) of the 6070 participants who did not report influenza vaccination said their decision was not influenced by the COVID-19 pandemic.

## Discussion

### Principal Findings

In this cohort of 11,237 adults with CVD, digital intervention was associated with a significantly higher rate of self-reported influenza vaccination at the end of the study period than control participants. Based on epidemiologic estimates, roughly 26 million Americans have CVD [26], and an increase in vaccination rates of 2.06% as shown in this trial would mean another 535,600 persons with CVD being immunized. This increase would likely translate to substantial reductions in morbidity, mortality, and costs to the health care system, as well as potential improvements in the quality of life if applied at scale.

Our findings add to a growing body of evidence that interventions delivered via digital communication channels can be effective in improving vaccination rates among high-risk patients. Previous randomized studies have generally shown significantly improved influenza vaccination rates with email prompts, app-based messages, SMS text messaging, and web-based interventions in general adult populations [14,15,23,27-31], high-risk patients (some of whom had heart disease) [16,32], and pregnant women [33-35], but a few have not [36-38]. To our knowledge, this is the first randomized study to show promising results with a digital intervention specifically designed for and delivered to a population with CVD.

The patient-centered digital interventions, developed with evidence-based behavioral theory of vaccine behavior [19], were generally viewed as informative, trustworthy, and engaging. In all, about 45% of the intervention group completed all intervention messages, compared to 27% in our previous study [16] and the industry standard of 22% [39], indicating very strong engagement. These messages also produced results at least as good as other recent app-based digital influenza vaccination interventions in general Canadian [13] and US adult populations [14,15].

Older age, more education, and White or Asian race were significant predictors of vaccination in this study. The apparent lack of effect in other participants of color might reflect small sample sizes or heterogeneity among non-White participants. Further assessment of racial and ethnic differences in responses to nontailored digital interventions is needed. This study does reinforce the importance of engagement with the health care system, as participants who saw a health care provider, were told they were in a high-risk group, or were offered the influenza vaccine were more likely to report vaccination.

Vaccination against influenza is a cost-effective method for reducing some of the risk associated with CVD [40,41]. Combined with the cost-effectiveness of digital intervention design and deployment relative to other prevention strategies, the messaging presented here appears to be suitable as a population-health management strategy in the context of limited budgets for health systems, insurers, and public agencies. In addition, half of the 380,000 people [1] hospitalized annually with influenza in the United States have heart disease [5]. Scaling this digital intervention to the larger population of people with CVD could help reduce hospitalizations and emergency department and clinic visits, along with days of productivity lost, particularly in already digitally connected populations.

The strengths of this study include its decentralized, pragmatic nature, which can provide high-quality evidence of effectiveness in real-world settings. Other strengths imclude its large sample size, nationwide scope, and variety of data collected, including patient-generated health data. The study also reflects real-world data on vaccination rates among persons with variable risk levels from influenza infection conferred by different cardiovascular diagnoses. The design of the study may inspire the design of future vaccination campaigns to assess the drivers of vaccination and their public health impact and investigate vaccination behavior in other patient populations.

### Limitations and Future Work

Participants reported their CVD diagnoses at different times via different survey sources (eg, historical surveys vs current self-reports). This method resulted in discrepancies from using different data sources in health outcomes, potentially due to question formatting and the time period for recall: almost a third of participants reported having none of the candidate CVD conditions despite previous self-reports of such disease. Future studies could forgo blinding in favor of supplementing self-reports with additional sources of information (eg, health claims and medical records). Participants were blinded to participation, reflecting real-world engagement with health messages outside of a known research-related setting. The potential influence of unknowingly participating in research is unclear. Only about 23% of the sample reported on the primary end point. The generalizability of findings to nonresponders is therefore limited. We also have no knowledge about why participants did not respond.

This trial was conducted during the COVID-19 pandemic. Due to the pandemic, participants may have had increased awareness of viral diseases and vaccines generally through other sources (eg, governmental sources, television, and social media), possibly limiting the generalizability of our findings, although most participants in both groups stated that the pandemic did not affect their decision about influenza vaccination.

Participants in the intervention group were compensated in the form of points, which could be redeemed for cash. However, given that the total possible monetary compensation was only US $1.52 regardless of vaccination status, it likely did not influence the motivation to vaccinate enough to impact the outcome.

All participants were existing members of the Achievement platform, reflecting a population already engaged with digital technology. The baseline (control) vaccination rate (59.25%) was also about 8% higher than the 51% CDC average for individuals with comorbidities [7]. Thus, it might have been more difficult to see an incremental uplift compared to populations with less technology use or a lower baseline vaccination rate.

Most of the population was female, non-Hispanic, and White. The effects of the intervention in other demographic groups are less certain, although the sample size was sufficient for models adjusting for age, education, sex, and race to confirm that the intervention effect remained significant. Barriers to health equity in accessing digital health interventions and methodologies remain significant [42]. This study should serve as a foundation for future evaluation and tailoring to reach individuals from diverse backgrounds more effectively, as Brewer and colleagues [43] have shown that people from diverse racial and ethnic backgrounds engage with digital health information via the web and digital health research at a high rate.

Although several evidence-based sources and techniques were leveraged in the development of the intervention messages, their exact mechanisms of action are unknown. The act of prompting, rather than the content, might result in similar improvement. Future studies examining which components or messages would be the most beneficial could help optimize future interventions while minimizing burden.

### Conclusions

A digital intervention using health condition–relevant information and widely available public health information can be an effective way to increase influenza vaccination rates in persons with CVD. These results may have broader public health implications as an easily scalable intervention to increase vaccination behavior. Future studies should examine the effectiveness and cost-effectiveness of such digital campaigns in diverse populations with other chronic conditions and for other types of vaccination, such as COVID-19 vaccines.

## Appendix

Multimedia Appendix 1 Supplemental information.

Multimedia Appendix 2 CONSORT (Consolidated Standards of Reporting Trials) eHealth checklist.

## Abbreviations

CDC: Centers for Disease Control and Prevention
CVD: cardiovascular disease
OR: odds ratio

## Data Availability

Qualified researchers may request access to the aggregate results and related study documents including the study report, study protocol with any amendments, blank case report form, statistical analysis plan, and data set specifications. Further details on Sanofi’s data sharing criteria, eligible studies, and process for requesting access can be found at https://www.vivli.org/.
