# Supplementary material for: Influence of Digital Intervention Messaging on Influenza Vaccination Rates Among Adults With Cardiovascular Disease in the United States: Decentralized Randomized Controlled Trial
Source: J Med Internet Res. 2022 Oct 7;24(10):e38710. doi: 10.2196/38710 (PMC9587491; doi:10.2196/38710)
Supplement: Multimedia Appendix 1 [file jmir_v24i10e38710_app1.pdf]

Supplement, Marshall N et al. Influence of Digital Intervention Messaging on Influenza Vaccination Rates Among Adults with Cardiovascular Disease in the U.S.: A Decentralized Randomized Clinical Trial

Digital CVD Intervention Development

The digital intervention messages (Supplement Table 1) were developed using a 3-part approach [18], building on a previous study [16] and the Theory of Planned Behavior [19]. First, researchers surveyed 844 participants with CVD, to assess their behaviors, thoughts, and beliefs surrounding influenza vaccination. Second, researchers conducted semistructured interviews with 23 of the participants who expressed vaccine hesitancy, exploring perceptions around vaccination and reactions to message themes and designs. Interviews were conducted in 2 phases: the first focused on high-level message themes and delivery mechanisms, and the second focused on wording and design details. Interviewers identified key design implications from prominent patient perceptions to draft message themes and content. Intervention message designs were refined throughout the interviewing process using Rapid Iterative Testing and Evaluation (RITE)-inspired methods [20].

Third, between interview phases, researchers convened an expert panel of cardiologists, behavioral scientists, and vaccine policy specialists for input on the messages. This approach to intervention development was consistent with the Patient Centered Outcomes Research Institute (PCORI) recommendation for developing interventions to increase participant recruitment and retention, improve patient outcomes, and increase research validity and relevance to the real world [21].

Each intervention message provided informational content on the influenza vaccine (sourced from health experts such as the CDC and the American Heart Association and emphasizing relationships between influenza and CVD) and behavioral prompts (e.g., reminders or encouragement) related to influenza vaccination. Participants were rewarded with Achievement points for completing calls to action; 100 points was convertible to a financial incentive of $0.10, which could be redeemed as monetary compensation or donated to charitable organizations once a threshold of $10 was reached. Fifty points were awarded per intervention message completed, 3 points per optional informational email requested, and 300 points per survey completed. The maximum number of points possible was 1,518, valued at $1.52.

Table S1. Digital intervention message content.

Message	Actions to Take	Information and Content Conveyed	Theory of Planned Behavior Factor[3]
1. Videos: Flu and Chronic Health Conditions	• Watch a video<br>• Receive optional follow-up email with videos	• Perspectives from the Mayo clinic and a patient with a chronic condition about why the flu shot is important	Utility, risk perception, knowledge, attitude, cues to action

2. Quiz: Flu Shot IQ	● Take an interactive quiz<br>● Receive optional follow-up email with information referenced within the quiz	● Misconceptions about the flu shot<br>● Information on flu-related complications and higher risks for people with CVD	Utility, risk perception, knowledge, experience, social benefit, cues to action
3. Article: Flu Shot Cost	● Read article (click "done" at end of article)<br>● Receive optional follow-up email with the article	● Potential cost at different organizations (with and without insurance)<br>● Brief message about flu and heart	Utility, knowledge, context determinant (access, cues to action), social benefit
4. Article: Flu and Heart Disease	● Read article (click "done" at the end of the article)<br>● Receive optional follow-up email with the article	● Increased risks of flu-related complications for people with CVD	Utility, risk perception, knowledge, social benefit, experience, cues to action
5. Location Identifier	● Input ZIP code to identify nearby locations<br>● Receive optional follow-up email with HealthMap tool to identify locations	● Nearby locations to get the flu shot<br>● Brief message about importance of vaccination during COVID-19 pandemic	Perceived behavioral control, context determinant (access, cues to action)
6. Date Picker with Reminder Emails	● Pick a date and schedule optional reminders<br>● Receive optional follow-up email with referenced information about heart attack risk<br>● Receive reminders to get the flu shot	● Brief message about increased risk of heart attack after influenza	Perceived behavioral control, utility, context determinant (access, cues to action)

Abbreviations: CVD, cardiovascular disease.

Figure S1. Screenshots of digital intervention messaging.

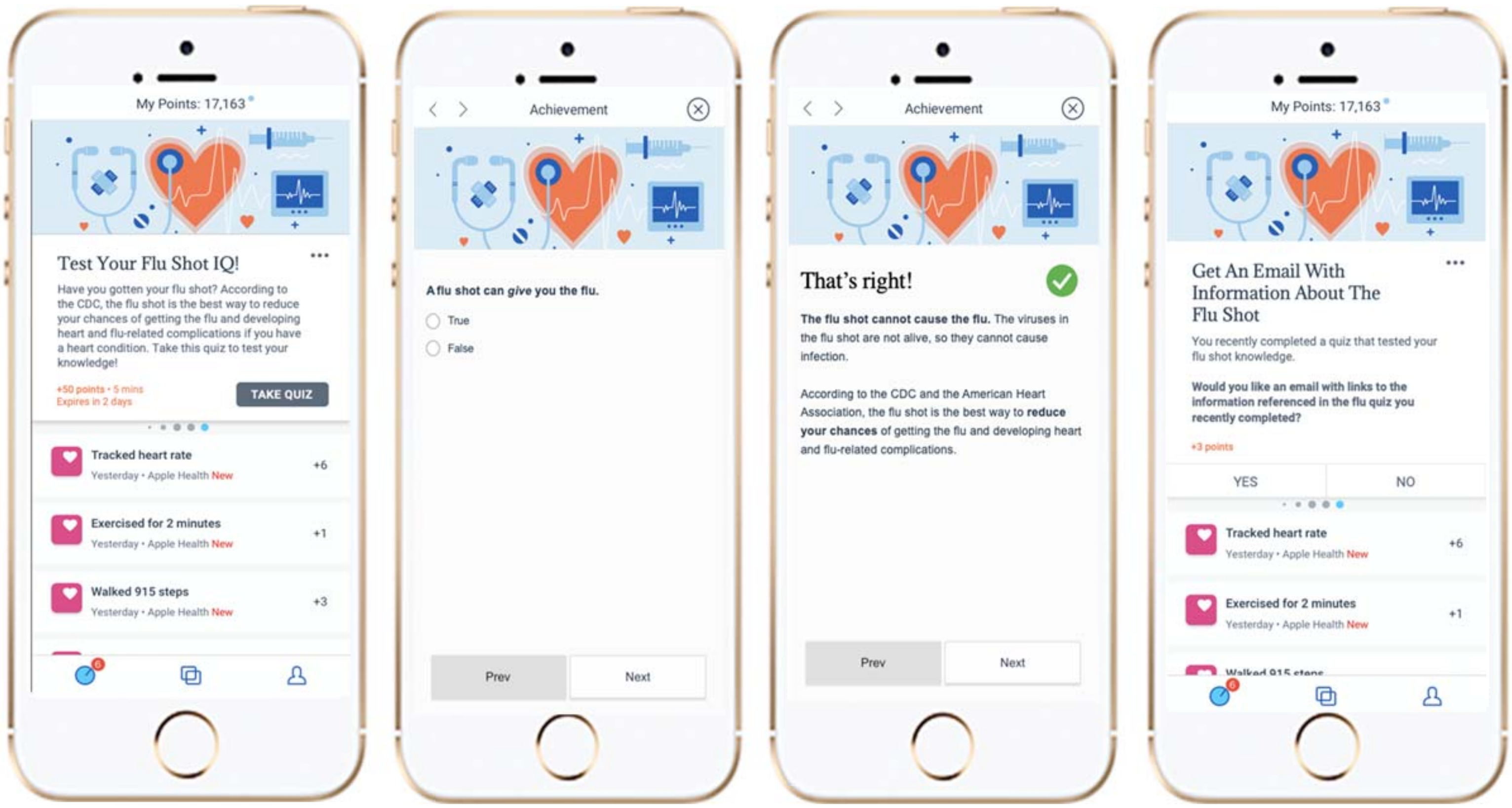

Message "offers" invited people with CVD to engage with the messages. Messages were delivered via the Achievement application (app); as all participants were recruited from the app they were familiar with the interface. All offers were designed to provide concise and engaging information that highlights message importance before inviting people to click into the message itself. Participants clicked into the message directly from the offer to view the content. All messages concluded with the ability to receive an email with the information or resources presented in the message.

Figure S2. Message linked to two videos discussing the need to get the influenza vaccination: one from a doctor's perspective and another from a patient's.

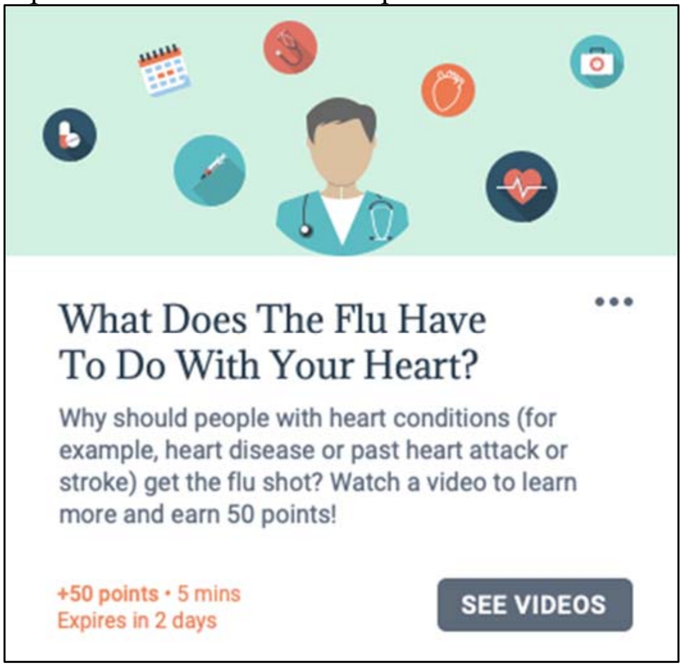

Figure S3. Message invited people to take an interactive quiz to test their knowledge about the influenza vaccine, providing feedback on their responses.

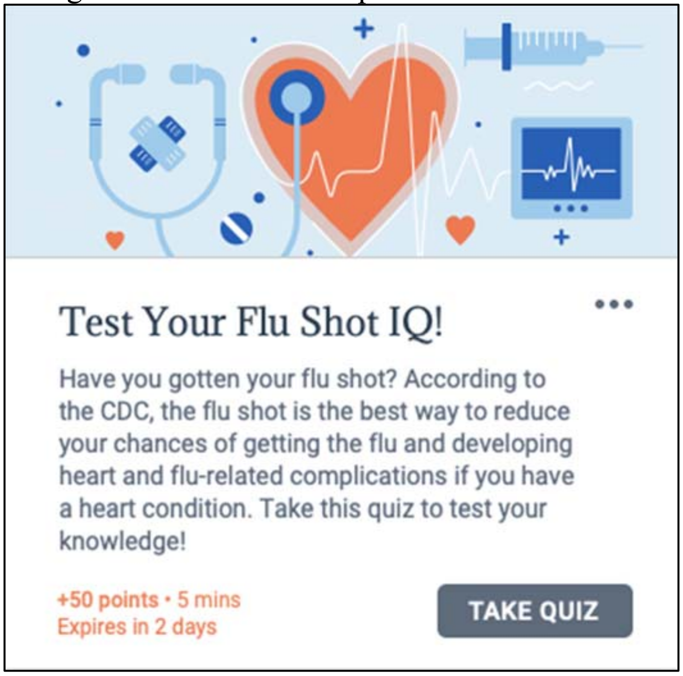

Figure S4. Message linked to an article that describes possible places to get the influenza vaccine and what the vaccine might cost at each place.

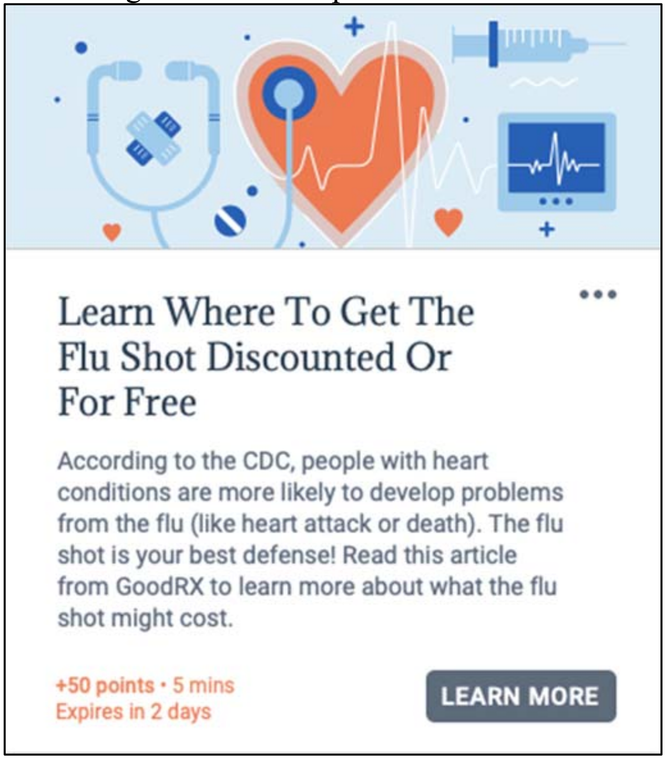

Figure S5. Message linked to an article with detailed information on the relationship between influenza and CVDs.

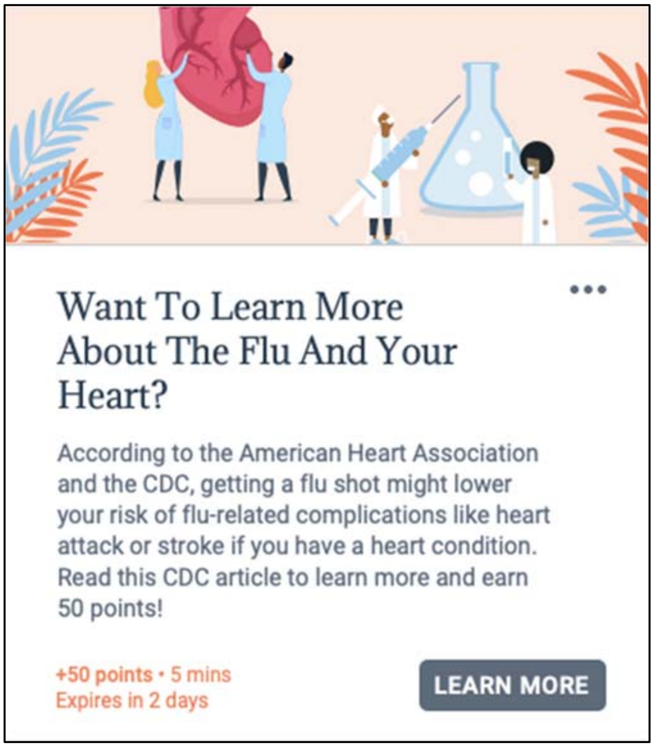

Figure S6. Message provided information on nearby locations to get the flu shot using the vaccinefinder.org tool. The offer also incorporated information about COVID-19.

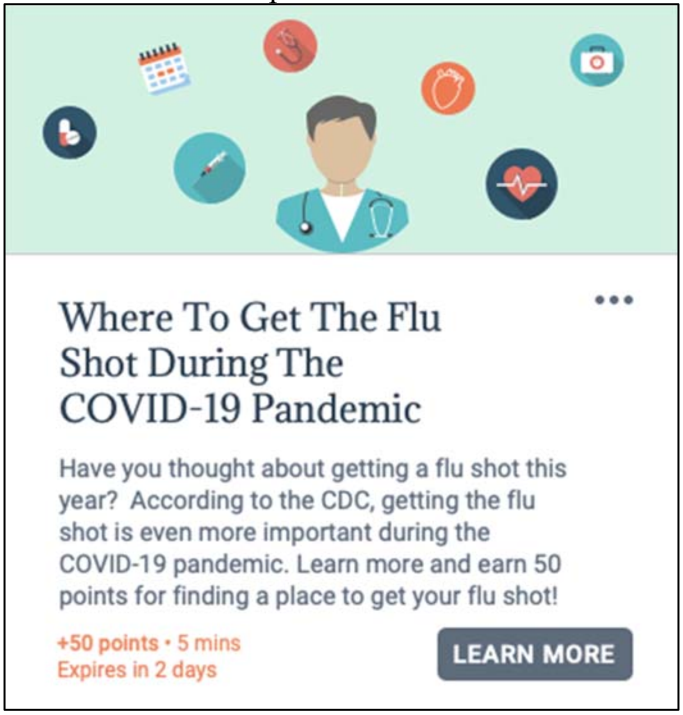

Figure S7. Message asked people to pick a date to get vaccinated and gave them an option of receiving email reminders to do so.

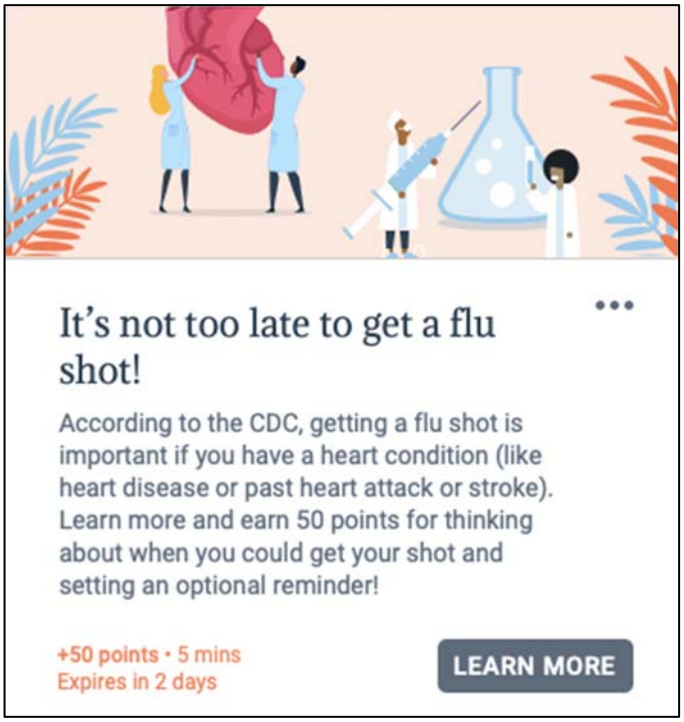

Figure S8. Geographic distribution of study participants.

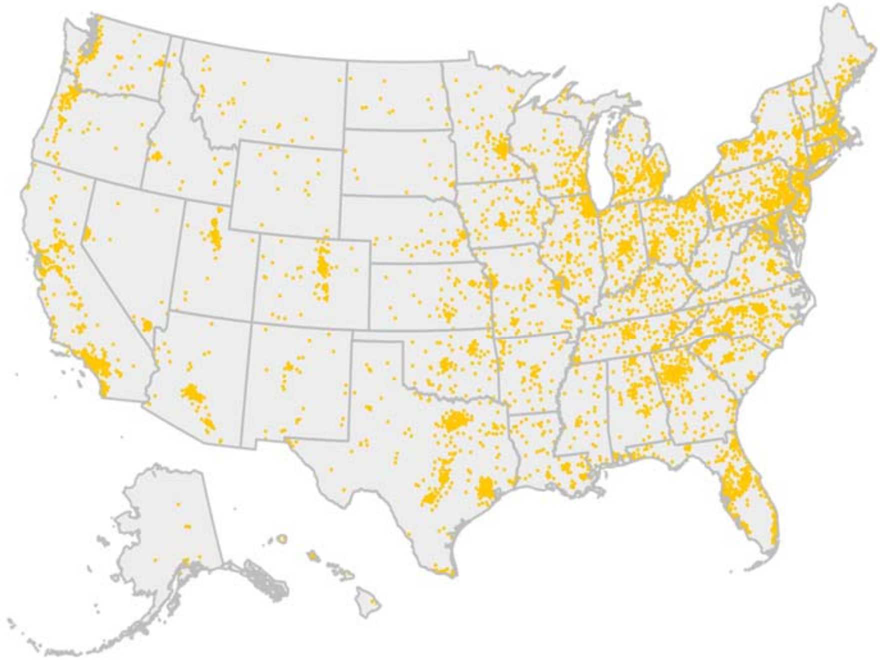

Figure S9. SHapley Additive exPlanations (SHAP) values for predictors of self-reported influenza vaccination.

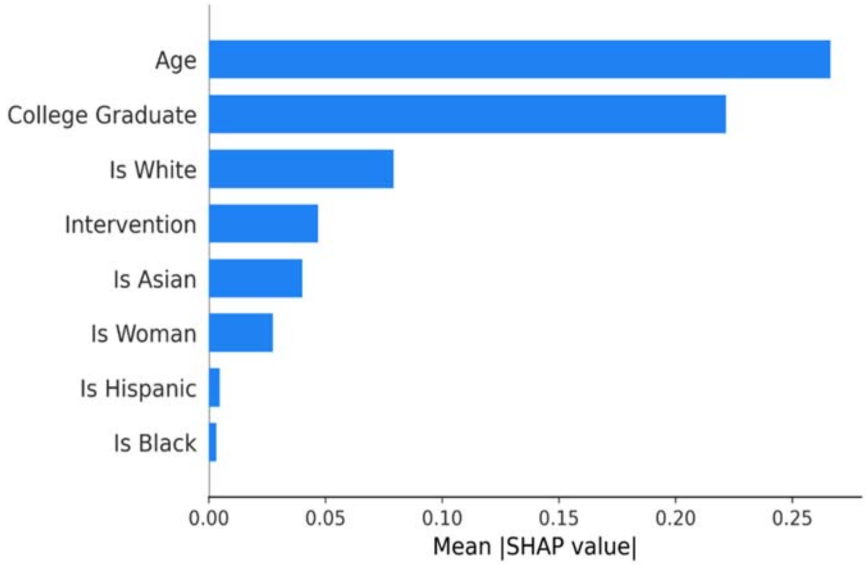

Figure S10. Self-reported vaccination rates by white vs. other race.

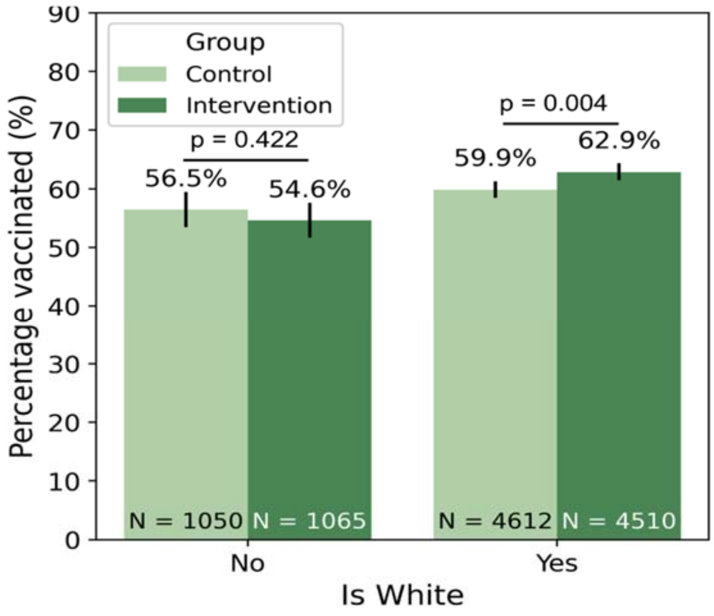

Figure S11. Digital intervention message preferences.

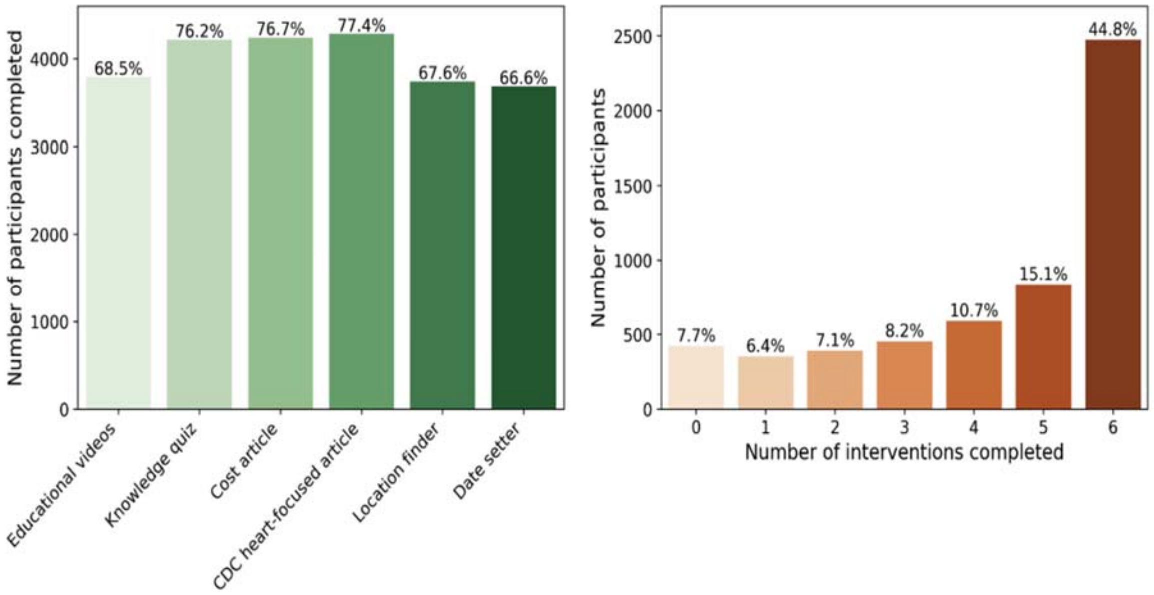

Figure S12. Vaccine drivers and barriers, regression over all study participants. All P=0.

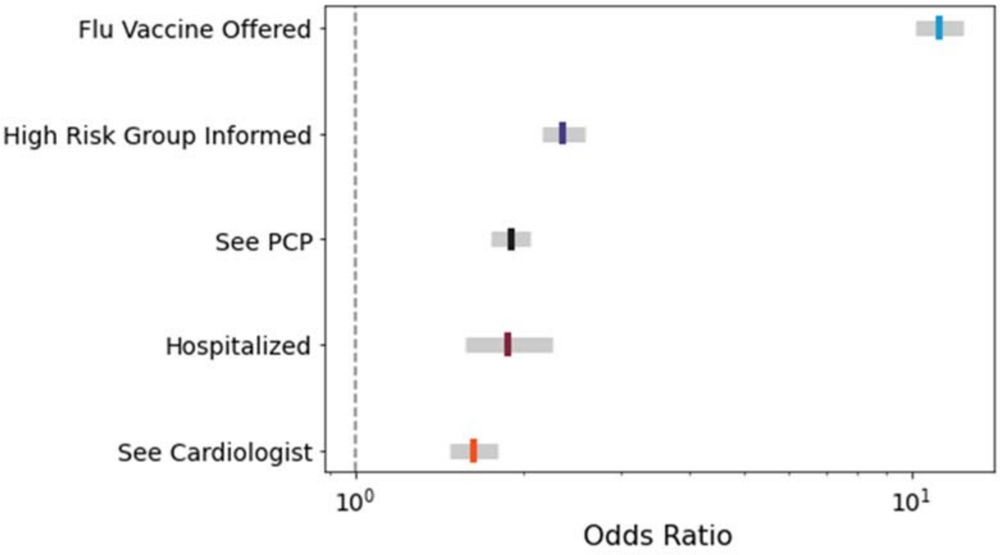

Figure S13. Impact of COVID-19 diagnosis on influenza vaccination.

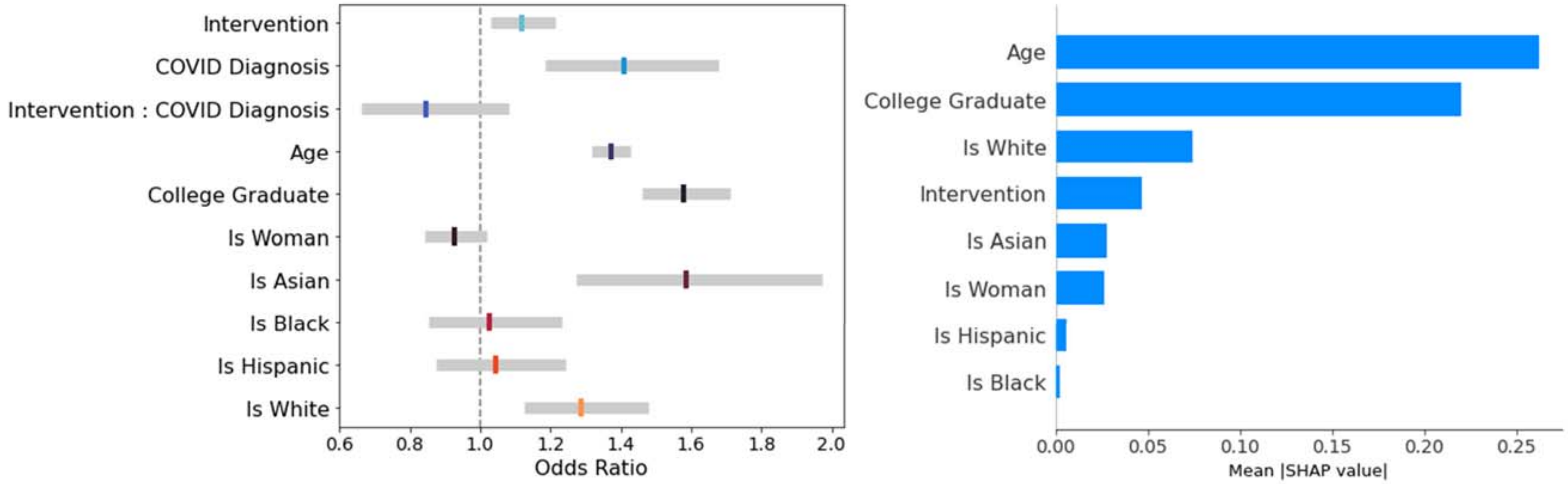

Variable	*P* value
Intervention	.093
COVID Diagnosis	<.001
Intervention:COVID Diagnosis	.485
Age	<.001
College Graduate	<.001
Is Woman	.330
Is Asian	.002
Is Black	.997
Is Hispanic	.665
Is White	<.001
