# Supplementary material for: Influence of Digital Intervention Messaging on Influenza Vaccination Rates Among Adults With Cardiovascular Disease in the United States: Decentralized Randomized Controlled Trial
Source: J Med Internet Res. 2022 Oct 7;24(10):e38710. doi: 10.2196/38710 (PMC9587491; doi:10.2196/38710)
Supplement: Multimedia Appendix 2 [file jmir_v24i10e38710_app2.pdf]

CONSORT-EHEALTH Checklist V1.6.2 Report	Manuscript Number	38710
**(based on CONSORT-EHEALTH V1.6), available at [http://tinyurl.com/consort-ehealth-v1-6].**		
**Date completed**		
4/19/2022 10:39:29		
**by**		
Nell Marshall		
Influence of Digital Intervention Messaging on Influenza Vaccination Rates Among Adults With Cardiovascular Disease in the United States: A Decentralized Randomized Clinical Trial		
TITLE		
**1a-i) Identify the mode of delivery in the title**		
"Influence of Digital Intervention Messaging on Influenza Vaccination Rates Among Adults With Cardiovascular Disease in the United States: A Decentralized Randomized Clinical Trial"		
**1a-ii) Non-web-based components or important co-interventions in title**		
not applicable; no non-web-based components or cointerventions were used		
**1a-iii) Primary condition or target group in the title**		
"Influence of Digital Intervention Messaging on Influenza Vaccination Rates Among Adults With Cardiovascular Disease in the United States"		
ABSTRACT		
**1b-i) Key features/functionalities/components of the intervention and comparator in the METHODS section of the ABSTRACT**		
" Adults with self-reported cardiovascular disease who were members of the Achievement platform were randomized to receive or not receive a series of 6 patient-centered digital intervention messages promoting influenza vaccination. "		
**1b-ii) Level of human involvement in the METHODS section of the ABSTRACT**		
Not applicable; no human involvement in the study.		
**1b-iii) Open vs. closed, web-based (self-assessment) vs. face-to-face assessments in the METHODS section of the ABSTRACT**		
"This was a randomized, controlled, single-blind decentralized trial conducted at individual locations throughout the United States over the 2020-2021 influenza season. Adults with self-reported cardiovascular disease who were members of the Achievement platform were randomized to receive or not receive a series of 6 patient-centered digital intervention messages promoting influenza vaccination. The primary endpoint was the between-group difference in self-reported vaccination rates at 6 months after randomization."		
**1b-iv) RESULTS section in abstract must contain use data**		
"Of the 49,138 randomized participants, responses on the primary endpoint were available for 11,237 (23%; 5575 in the intervention group and 5662 in the control group). The vaccination rate was significantly higher in the intervention group than among controls: 3418/5575 (61.31%) vs. 3355/5662 (59.25%), respectively; relative risk 1.03 [95% CI 1.004-1.066]; P=.027). "		
**1b-v) CONCLUSIONS/DISCUSSION in abstract for negative trials**		
"This personalized, evidence-based digital intervention was effective in increasing vaccination rates in this population of high-risk people with cardiovascular disease."		
INTRODUCTION		
**2a-i) Problem and the type of system/solution**		
See the first three paragraphs of the Introduction, plus: "The primary objective of this study was to examine the efficacy of a digital intervention designed to increase self-reported influenza vaccination rates in individuals with CVD."		
**2a-ii) Scientific background, rationale: What is known about the (type of) system**		
"Novel, scalable, cost-optimal, and effective solutions are needed to address barriers to influenza vaccination among people with CVD, such as complacency, time/cost constraints, and lack of confidence [12]. Observational [13] and randomized controlled trials [14, 15] have shown the effectiveness of digital messaging to increase vaccine uptake in general adult populations. In a randomized trial of digital messaging in persons with diabetes, a population also at increased risk of influenza-related complications [16], the vaccination rate was 3.1% higher in the intervention group compared with controls. "		
**Does your paper address CONSORT subitem 2b?**		
"The primary objective of this study was to examine the efficacy of a digital intervention designed to increase self-reported influenza vaccination rates in individuals with CVD. "		
METHODS		
**3a) CONSORT: Description of trial design (such as parallel, factorial) including allocation ratio**		
"This 8-month pragmatic randomized controlled trial was conducted virtually in the United States. Participants were blinded to study participation status to minimize observation bias, though all participants agreed that their survey responses and behavioral data would be used for research purposes before completing each survey..." "Evidation Health ... enrolled participants, and randomized them ... into the intervention or control group ..."		
**3b) CONSORT: Important changes to methods after trial commencement (such as eligibility criteria), with reasons**		
Not applicable; no important changes after trial commencement		
**3b-i) Bug fixes, Downtimes, Content Changes**		
Not applicable; no important changes made on the intervention messages during the trial		
**4a) CONSORT: Eligibility criteria for participants**		
"Eligible participants were at least 18 years old, living in the United States, with any of the following self-reported on the Achievement platform (eg, through past surveys): atrial fibrillation; abnormal or irregular heart rhythm or other arrhythmic heart disease; cardiac arrest or myocardial infarction; coronary artery disease treated with medication, stenting, percutaneous intervention, or bypass surgery; congestive heart failure; or stroke or cerebrovascular accident."		
**4a-i) Computer / Internet literacy**		
"All participants were existing members of the free Achievement platform, reflecting a population already engaged with digital technology." "Thus it might have been more difficult to see an incremental uplift compared with populations with less technology use..."		
**4a-ii) Open vs. closed, web-based vs. face-to-face assessments:**		
"This 8-month pragmatic randomized controlled trial was conducted virtually..." "All participants were members of the free Achievement mobile health and research platform..." "Participants took no action to enroll and were not informed about their participation status." It is theoretically possible that a user could have multiple identities, but we did not investigate this possibility.		
**4a-iii) Information giving during recruitment**		
"Participants took no action to enroll and were not informed about their participation status." "Because the digital intervention messages were consistent with publicly available information on influenza vaccination, researchers obtained a waiver of informed consent from this IRB on the basis that participants would face only minimal risk from the study. Participants were informed about how their survey responses and behavioral data would be used through a Data Usage and Permissions Agreement."		
**4b) CONSORT: Settings and locations where the data were collected**		
"Participants were asked to complete online surveys at baseline, 3 months ..., and 6 months ..." "Participants self-reported their vaccination status (Yes or No) via the app at baseline, 3 months, and 6 months. Participants also reported the estimated date of vaccination, if any, on the 3- and 6-month surveys."		
**4b-i) Report if outcomes were (self-)assessed through online questionnaires**		
""Participants self-reported their vaccination status (Yes or No) via the app at baseline, 3 months, and 6 months. Participants also reported the estimated date of vaccination, if any, on the 3- and 6-month surveys."		
**4b-ii) Report how institutional affiliations are displayed**		
No institutional affiliations were included in the digital intervention messages.		
**5) CONSORT: Describe the interventions for each group with sufficient details to allow replication, including how and when they were actually administered**		
**5-i) Mention names, credential, affiliations of the developers, sponsors, and owners**		
"This sponsor was involved in intervention development, study design, data interpretation, and writing of the report, and was kept informed during data collection and data analysis." "NM, JLL, J. Schroeder, WNL, and J. See are employees of Evidation Health, Inc., the developer of the Achievement health and research platform, and may hold stock options in Evidation Health." "MG, M. Mercer, SS, and JL are employees of Sanofi, the study sponsor. JL and SS are also shareholders of Sanofi."		
**5-ii) Describe the history/development process**		
See the first paragraph in the Supplement.		
**5-iii) Revisions and updating**		
Not applicable; the digital intervention messages did not change during the trial		
**5-iv) Quality assurance methods**		
Supplement: "Each intervention message provided informational content on the influenza vaccine (sourced from health experts such as the CDC and the American Heart Association and emphasizing relationships between influenza and CVD...)"		
**5-v) Ensure replicability by publishing the source code, and/or providing screenshots/screen-capture video, and/or providing flowcharts of the algorithms used**		
Screenshots of the intervention and the content of the digital intervention messages are provided in the Supplement.		

**5-vi) Digital preservation**		
The digital intervention messages were available only during the period of the trial. Screenshots of the intervention are provided in the Supplement.		
**5-vii) Access**		
"All participants were members of the free Achievement mobile health and research platform... " "Eligible participants were at least 18 years old, living in the United States, with any of the following self-reported on the Achievement platform..." The Supplement details the reward structure for completing messages: "The maximum number of points possible was 1,518, valued at $1.52."		
**5-viii) Mode of delivery, features/functionalities/components of the intervention and comparator, and the theoretical framework**		
"The 6 digital intervention messages were developed using a 3-part approach ... See the Supplement for details of the development process and content of the intervention messages, which were delivered via the Achievement platform."		
**5-ix) Describe use parameters**		
Not applicable; digital intervention messages and surveys were sent at set intervals.		
**5-x) Clarify the level of human involvement**		
Not applicable; there was no human involvement with the messages or surveys.		
**5-xi) Report any prompts/reminders used**		
"Reminder messages were used to motivate survey completion."		
**5-xii) Describe any co-interventions (incl. training/support)**		
Not applicable; there were no co-interventions.		
**6a) CONSORT: Completely defined pre-specified primary and secondary outcome measures, including how and when they were assessed**		
See the Primary and Other Outcomes section of the Methods for definitions.		
**6a-i) Online questionnaires: describe if they were validated for online use and apply CHERRIES items to describe how the questionnaires were designed/deployed**		
The surveys did not undergo validation for this trial. All surveys were deployed via the Achievement platform. "Participants were asked to complete online surveys at baseline, 3 months ... and 6 months... Reminder messages were used to motivate survey completion." "Participants self-reported their vaccination status ... via the app at baseline, 3 months, and 6 months. Participants also reported the estimated date of vaccination, if any, on the 3- and 6-month surveys." "Each survey measured drivers and barriers to vaccination as well as vaccine knowledge." These are defined in the Methods.		
**6a-ii) Describe whether and how "use" (including intensity of use/dosage) was defined/measured/monitored**		
"To assess engagement with the intervention messages, we examined platform-generated data indicating that the person had completed a given message, and created a summary measure indicating the number of messages completed."		
**6a-iii) Describe whether, how, and when qualitative feedback from participants was obtained**		
No qualitative feedback was collected during the trial.		
**6b) CONSORT: Any changes to trial outcomes after the trial commenced, with reasons**		
"Participants were asked to complete online surveys at baseline, 3 months ..., and 6 months ..." "Participants self-reported their vaccination status (Yes or No) via the app at baseline, 3 months, and 6 months. Participants also reported the estimated date of vaccination, if any, on the 3- and 6-month surveys."		
**7a) CONSORT: How sample size was determined**		
**7a-i) Describe whether and how expected attrition was taken into account when calculating the sample size**		
See the Sample Size Calculation section of the manuscript.		
**7b) CONSORT: When applicable, explanation of any interim analyses and stopping guidelines**		
See the Primary and Other Outcomes section of the Methods for definitions.		
**8a) CONSORT: Method used to generate the random allocation sequence**		
See the Randomization and Blinding section of the manuscript.		
**8b) CONSORT: Type of randomisation; details of any restriction (such as blocking and block size)**		
See the Randomization and Blinding section of the manuscript.		
**9) CONSORT: Mechanism used to implement the random allocation sequence (such as sequentially numbered containers), describing any steps taken to conceal the sequence until interventions were assigned**		
Randomization was done by block randomization on each of the 6 cardiovascular conditions we included.		
**10) CONSORT: Who generated the random allocation sequence, who enrolled participants, and who assigned participants to interventions**		
"Evidation Health generated the random allocations, enrolled participants, and randomized them using block randomization ... into the intervention or control group ..."		
**11a) CONSORT: Blinding - If done, who was blinded after assignment to interventions (for example, participants, care providers, those assessing outcomes) and how**		
**11a-i) Specify who was blinded, and who wasn't**		
"This was a randomized, controlled, single-blind decentralized trial ..." "Participants were blinded to study participation status to minimize observation bias..."		
**11a-ii) Discuss e.g., whether participants knew which intervention was the "intervention of interest" and which one was the "comparator"**		
Not applicable; informed consent was waived for the trial and participants were blinded to their participation status.		
**11b) CONSORT: If relevant, description of the similarity of interventions**		
"Participants were randomized into either the intervention group, which received the digital intervention messages, or the control group, which received none of the messages."		
**12a) CONSORT: Statistical methods used to compare groups for primary and secondary outcomes**		
"We first compared the unadjusted proportions of participants reporting vaccination at follow-up between the intervention and control groups." See also the last paragraph of the Methods.		
**12a-i) Imputation techniques to deal with attrition / missing values**		
Outcomes were computed based on the available data, and no imputation techniques were used.		
**12b) CONSORT: Methods for additional analyses, such as subgroup analyses and adjusted analyses**		
See the first two and the last paragraphs of the Methods.		
RESULTS		
**13a) CONSORT: For each group, the numbers of participants who were randomly assigned, received intended treatment, and were analysed for the primary outcome**		
See the first paragraph of the Results.		
**13b) CONSORT: For each group, losses and exclusions after randomisation, together with reasons**		
See Figure 1 in the manuscript.		
**13b-i) Attrition diagram**		
See Figure 1 in the manuscript.		
**14a) CONSORT: Dates defining the periods of recruitment and follow-up**		
See the first paragraph of the Results.		
**14a-i) Indicate if critical "secular events" fell into the study period**		
No critical secular events occurred during the trial.		
**14b) CONSORT: Why the trial ended or was stopped (early)**		
Not applicable; the trial was not stopped early.		
**15) CONSORT: A table showing baseline demographic and clinical characteristics for each group**		
See Table 1 in the manuscript.		
**15-i) Report demographics associated with digital divide issues**		
Our population was a selected group already using the app platform. This is acknowledged in the Limitations.		
**16a) CONSORT: For each group, number of participants (denominator) included in each analysis and whether the analysis was by original assigned groups**		
**16-i) Report multiple "denominators" and provide definitions**		
In this trial, if participants provided vaccination status at 3 months or 6 months, they were included in the analysis (thus generating the denominators, which are included in the Results).		
**16-ii) Primary analysis should be intent-to-treat**		
If participants provided vaccination status at 3 months or 6 months, they were included in the analysis (thus generating the denominators). The fact that this was a selected population is acknowledged in the Limitations.		
**17a) CONSORT: For each primary and secondary outcome, results for each group, and the estimated effect size and its precision (such as 95% confidence interval)**		
See the Primary Outcome and Secondary Outcomes sections of the manuscript.		
**17a-i) Presentation of process outcomes such as metrics of use and intensity of use**		
"To assess engagement with the intervention messages, we examined platform-generated data indicating that the person had completed a given message, and created a summary measure indicating the number of messages completed."		
**17b) CONSORT: For binary outcomes, presentation of both absolute and relative effect sizes is recommended**		
See the Primary Outcome section of the Results.		

**18) CONSORT: Results of any other analyses performed, including subgroup analyses and adjusted analyses, distinguishing pre-specified from exploratory**		
See the Secondary Outcomes section of the Results.		
**18-i) Subgroup analysis of comparing only users**		
Not applicable; only people who indicated vaccination status on the 3- or 6-month survey were included in the analyses.		
**19) CONSORT: All important harms or unintended effects in each group**		
"Information on safety and adverse events was not collected, given the minimal-risk nature of the intervention and study. "		
**19-i) Include privacy breaches, technical problems**		
There were no privacy breaches or technical problems.		
**19-ii) Include qualitative feedback from participants or observations from staff/researchers**		
This information was not collected.		
DISCUSSION		
**20) CONSORT: Trial limitations, addressing sources of potential bias, imprecision, multiplicity of analyses**		
**20-i) Typical limitations in ehealth trials**		
"The potential influence of unknowingly participating in research is unclear."<br>"All participants were existing members of the free Achievement platform, reflecting a population already engaged with digital technology. "<br>"...their exact mechanisms of action (of the intervention messages) are unknown. The act of prompting, rather than the content, might result in similar improvement."		
**21) CONSORT: Generalisability (external validity, applicability) of the trial findings**		
**21-i) Generalizability to other populations**		
See the Limitations section of the manuscript.		
**21-ii) Discuss if there were elements in the RCT that would be different in a routine application setting**		
The decentralized, pragmatic nature of this trial mimics a real-world setting. There was no human involvement, training sessions, or co-interventions, so adoption might not differ substantially.		
**22) CONSORT: Interpretation consistent with results, balancing benefits and harms, and considering other relevant evidence**		
**22-i) Restate study questions and summarize the answers suggested by the data, starting with primary outcomes and process outcomes (use)**		
See the Principal Findings paragraph of the Discussion.		
**22-ii) Highlight unanswered new questions, suggest future research**		
"Further assessment of racial and ethnic differences in responses to non-tailored digital interventions is needed." "Future studies could forgo blinding in favor of supplementing self-reports with additional sources of information (eg, health claims, medical records)." "This study should serve as a foundation for future evaluation and tailoring to reach individuals from diverse backgrounds more effectively..."		
Other information		
**23) CONSORT: Registration number and name of trial registry**		
"ClinicalTrials.gov NCT04584645; https://clinicaltrials.gov/ct2/show/NCT04584645"		
**24) CONSORT: Where the full trial protocol can be accessed, if available**		
The protocol is not publicly available.		
**25) CONSORT: Sources of funding and other support (such as supply of drugs), role of funders**		
See the Acknowledgments section of the manuscript.		
**X26-i) Comment on ethics committee approval**		
"The trial protocol was approved by Solutions IRB, Yarnell, Arizona, and was registered at ClinicalTrials.gov (NCT04584645)."		
**x26-ii) Outline informed consent procedures**		
See the Ethics Approval section of the manuscript.		
**X26-iii) Safety and security procedures**		
All identifiable information about participants...were secured by Evidation Health in accordance with all applicable local and state laws, regulations, and IRB policies ... Data were transmitted using secure encrypted protocols and stored on encrypted disks on secure and hardened servers. Administrative access to these servers was limited to only the necessary IT staff at Evidation Health. ... Personally Identifiable Information (PII) was only accessible to a restricted set of individuals, and was only used to distribute study material and for participant support purposes.		
**X27-i) State the relation of the study team towards the system being evaluated**		
"NM, JLL, J. Schroeder, WNL, and J. See are employees of Evidation Health, Inc., the developer of the Achievement health and research platform..."		
